# Mitochondrial dysregulation and oxidative stress in patients with chronic kidney disease

**DOI:** 10.1186/1471-2164-10-388

**Published:** 2009-08-21

**Authors:** Simona Granata, Gianluigi Zaza, Simona Simone, Gaetano Villani, Dominga Latorre, Paola Pontrelli, Massimo Carella, Francesco Paolo Schena, Giuseppe Grandaliano, Giovanni Pertosa

**Affiliations:** 1 Renal, Dialysis and Transplant Unit-Department of Emergency and Transplantation, University of Bari, Italy; 2 Department of Medical Biochemistry, Biology & Physics, University of Bari, Italy; 3 Department of Biomedical Sciences, University of Foggia, Foggia, Italy; 4 Medical Genetics Service, "Casa Sollievo della Sofferenza", Hospital, IRCCS, San Giovanni Rotondo, Italy

## Abstract

**Background:**

Chronic renal disease (CKD) is characterized by complex changes in cell metabolism leading to an increased production of oxygen radicals, that, in turn has been suggested to play a key role in numerous clinical complications of this pathological condition. Several reports have focused on the identification of biological elements involved in the development of systemic biochemical alterations in CKD, but this abundant literature results fragmented and not exhaustive.

**Results:**

To better define the cellular machinery associated to this condition, we employed a high-throughput genomic approach based on a whole transcriptomic analysis associated with classical molecular methodologies. The genomic screening of peripheral blood mononuclear cells revealed that 44 genes were up-regulated in both CKD patients in conservative treatment (CKD, n = 9) and hemodialysis (HD, n = 17) compared to healthy subjects (HS, n = 8) (p < 0.001, FDR = 1%). Functional analysis demonstrated that 11/44 genes were involved in the oxidative phosphorylation system. Western blotting for COXI and COXIV, key constituents of the complex IV of oxidative phosphorylation system, performed on an independent testing-group (12 healthy subjects, 10 CKD and 14 HD) confirmed an higher synthesis of these subunits in CKD/HD patients compared to the control group. Only for COXI, the comparison between CKD and healthy subjects reached the statistical significance. However, complex IV activity was significantly reduced in CKD/HD patients compared to healthy subjects (p < 0.01). Finally, CKD/HD patients presented higher reactive oxygen species and 8-hydroxydeoxyguanosine levels compared to controls.

**Conclusion:**

Taken together these results suggest, for the first time, that CKD/HD patients may have an impaired mitochondrial respiratory system and this condition may be both the consequence and the cause of an enhanced oxidative stress.

## Background

Chronic kidney disease (CKD) is characterized by a progressive deterioration of renal function. CKD is a common condition that, according to the recent report of NHANES III, affects 7.7% of the US population [1]. Recently, an international consensus categorized CKD into five stages according to the glomerular filtration rate [2]. The reduction of renal function, particularly in the more advanced stages, has been associated to significant changes in energy metabolism, nitrogen balance, protein-energy malnutrition and insulin resistance and with a significant increase in the generation of reactive oxygen species [3-5].

Although many of these biochemical alterations can be improved by renal replacement treatments, including hemodialysis (HD) and peritoneal dialysis, these procedures do not reconstitute the normal body homeostasis [6]. In fact, it has been extensively reported that patients in renal replacement therapy develop a complex disease comprising partially treated uremia and ill effects of dialysis, such as fluctuation in the extracellular fluid volume, residual inorganic ion disturbances and exposure to bioincompatible materials [7,8].

Additionally, it is well known that during these treatments the interaction of peripheral blood mononuclear cells (PBMC) with bioincompatible dialysis devices causes their activation with consequent increased synthesis and release of pro-inflammatory cytokines [9-12], imbalance between pro- and anti-oxidant activities resulting in high oxidative stress [13,14] and immune system deregulation [15]. All together these conditions may lead to severe clinical complications including cardiovascular disease, atherosclerosis, anemia and malnutrition with a consequent low quality of life, high risk of hospitalization and short survival of this patients' population [16-19].

In the last decade, numerous studies have investigated the molecular triggers and biological key elements associated to the development of these clinical changes, but the complete picture of this process is still incomplete [20-22].

New strategies based on the combination of traditional molecular approaches (e.g., polymerase chain reaction, western blotting) and innovative high-throughput technologies have been proposed to address this issue. In particular, microarrays, largely employed in the screening of complex biological events, analyzing simultaneously thousand of genes, represent one of the most powerful and highly sensitive tool to investigate the potential molecular interactions and multi-factorial variables involved in biological processes [23,24].

To date, only few studies have employed this methodology to select the molecular fingerprints associated with the onset and progression of renal damage and to build models defining the mechanisms underlying severe clinical complication associated to CKD and dialysis therapy [25-27]. However, these studies have been performed on a relatively small number of patients and on limited gene datasets.

Therefore, the aim of the present study was to uncover, through a combined strategy based on an innovative high-throughput technology (microarray) and classical molecular methodologies, the mechanisms underlying alterations in cell metabolism featuring patients with CKD.

## Results

### Microarray analysis

**Figure 1:**
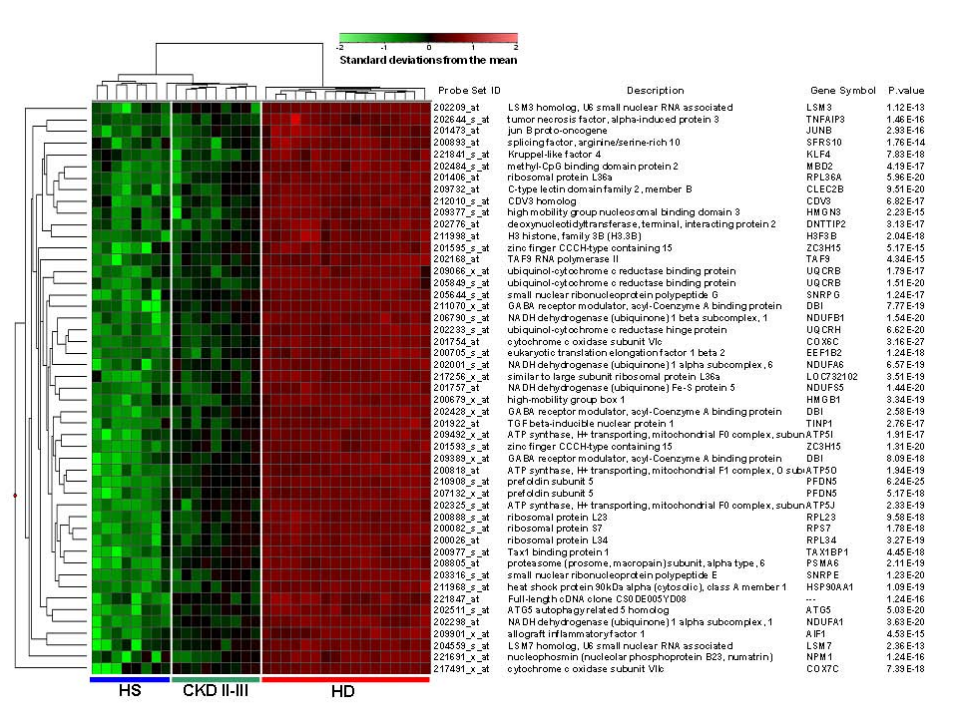
**"Supervised" hierarchical clustering discriminating healthy subjects (HS), chronic kidney disease (CKD) and hemodialysis (HD) patients**. (A) Patients are depicted as vertical columns, with blue line in the bottom indicating healthy subjects (HS) (n = 8), green indicating (CKD II–III) (n = 9) and red indicating (HD) (n = 17) patients. Forty-nine gene probe sets (rows, with gene names shown) were used for hierarchical clustering. P-values were calculated by ANOVA. The relative level of gene expression is depicted from lowest (green) to highest (red) according to the scale shown at the top.

To identify specific genomic fingerprints differentiating healthy subjects from those with chronic kidney disease, we analyzed the gene-expression profiling of PBMC isolated from 8 healthy subjects, 9 CKD patients on stage II–III (CKD II–III) and 17 patients undergoing HD treatment (HD). According to independent statistical algorithms and the estimated FDR, we identified 49 gene probe sets (corresponding to 44 genes) up-regulated in CKD II–III and HD and able to discriminate the three study groups (p < 0.001, FDR = 1%). However, we found only a slight and not significant difference in the genomic profile between CKD II–III and normal subjects (p < 0.06) (FIGURE 1). The latter result may be related to the low degree of renal failure of the CKD population included in the microarray analysis. The 2D hierarchical clustering using the 49 selected gene probe sets showed the degree of separation among the 3 study groups (FIGURE 1).

### Functional analysis of the transcriptomic profile identified by microarray

**Figure 2:**
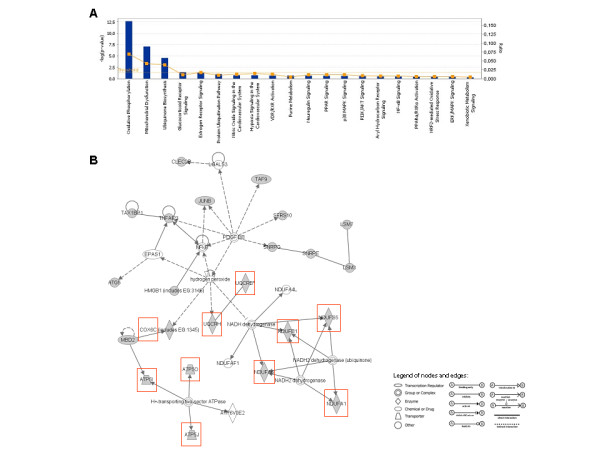
**Functional categorization, and Ingenuity Pathway Analysis (IPA) network of the top selected genes by microarray**. (A) Histogram represents the most significant canonical pathways generated using IPA software including the 44 genes discriminating the three study groups. Ratio was calculated by dividing the number of genes from our dataset that map to each single pathway by the total number of genes included into the canonical pathway. P-value was calculated using the right-tailed Fisher's Exact Test. Ratio and P-value are indicated on the left and right side of the histogram, respectively. (B) Network, algorithmically generated based on their functional and biological connectivity, was graphically represented as nodes (genes) and edges (the biological relationship between genes). Shaded nodes represented genes identified by our microarray analysis and others (empty nodes) were those that IPA automatically included because biologically linked to our genes based on evidence in the literature. Red shaded genes were those involved in oxidative phosphorylation system (OXPHOS).

**Figure 3:**
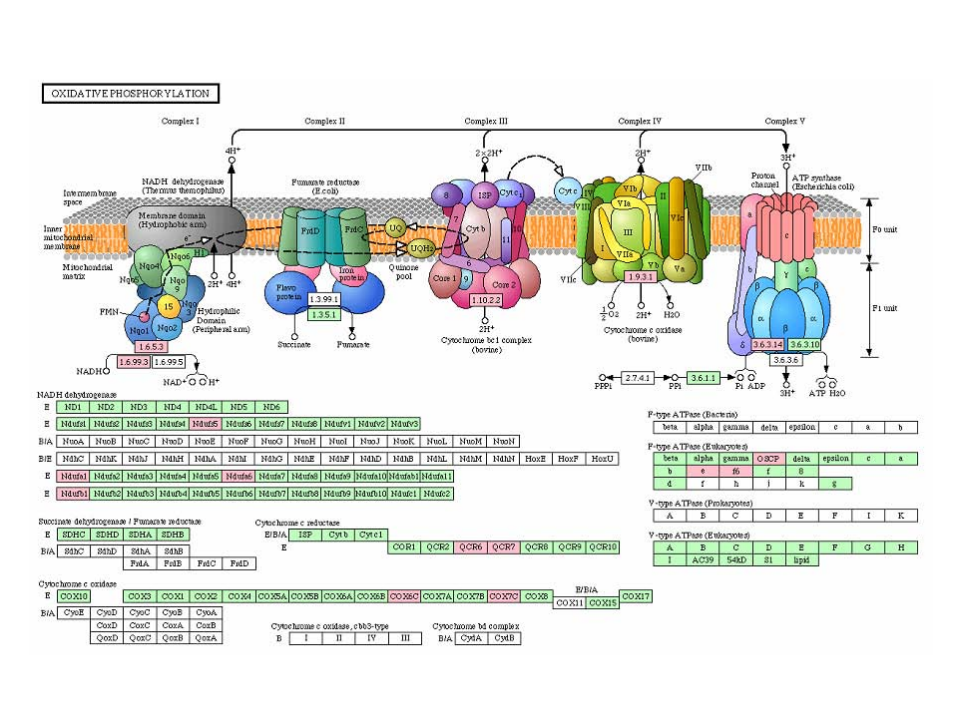
**KEGG pathway diagram including the 11 oxidative phosphorylation system (OXPHOS) genes selected by microarray analysis**. In the upper part are shown the five respiratory chain complexes with the corresponding E.C. numbers. In the bottom part, as rectangles, are indicated the subunits of each respiratory chain complex. The subunits encoded by genes selected by microarray analysis are shown by colored (pink shaded) rectangles.

Using Ingenuity Pathway Analysis (IPA) software, we found that the top selected genes were involved in several biological pathways including oxidative phosphorylation system, ubiquinone biosynthesis and glucocorticoids receptor signaling. However, oxidative phosphorylation system, including 11 of 44 genes (*ATP5O, COX6C, COX7C, NDUFS5, NDUFA6, UQCRH, NDUFA1, ATP5J, UQCRB, NDUFB1 *and *ATP5I*), reached the highest level of significance (FIGURE 2A). The relative connectivity diagram in Figure 2B shows the high degree of direct and indirect biological association (score = 30, p < 0.0001) among the selected genes. In addition, KEGG analysis reveals that the 11 genes encode for essential subunits of complex I, III, IV and V of oxidative phosphorylation pathway (FIGURE 3).

### Validation of microarray using 4 representative genes of the oxidative phosphorylation system

**Figure 4:**
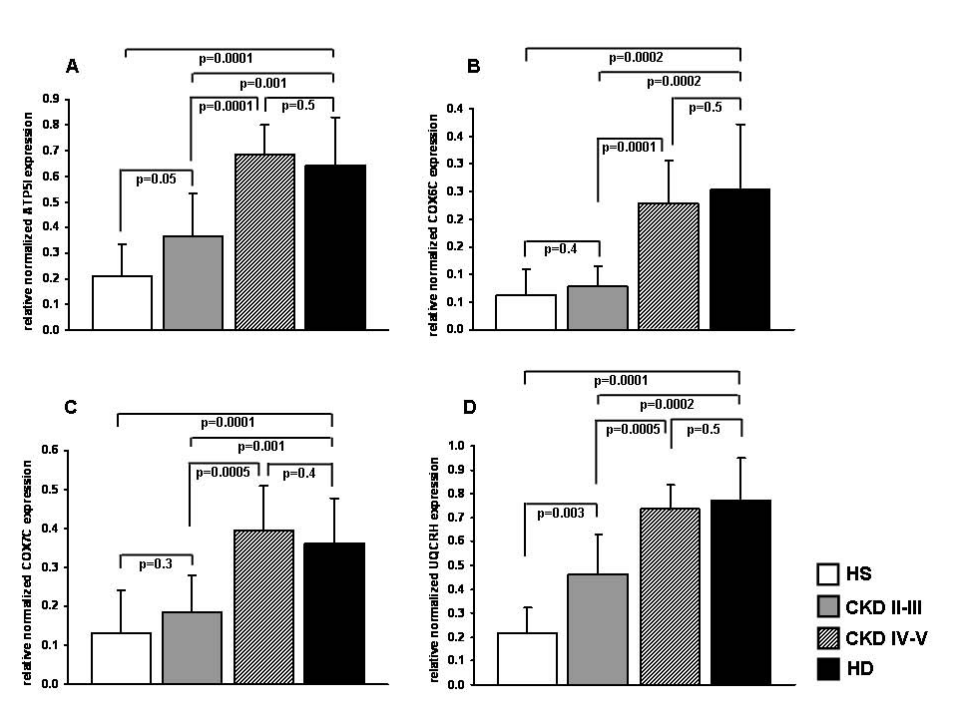
**ATP5I, COX6C, COX7C and UQCRH gene expression by RT-PCR in peripheral blood mononuclear cells (PBMC) from healthy subjects (HS), chronic kidney disease (CKD II–III and CKD IV–V) and hemodialysis (HD) patients**. The histograms represent the mean ± SD of ATP5I (A), COX6C (B), COX7C (C) and UQCRH (D) level of expression, determined by RT-PCR, in PBMC from 8 HS, 9 CKD II–III, 10 CKD IV–V and 30 HD patients. For all 4 genes, the expression level was significantly higher in HD and CKD IV–V compared to CKD II–III and HS.

*COX6C, COX7C, ATP5I*, and *UQCRH *mRNA levels, measured on the same microarray population and 10 additional CKD patients on stage IV–V, were significantly higher in HD and CKD IV–V compared to CKD II–III and healthy subjects. For all the genes analyzed there was no statistical difference in the expression levels between CKD IV–V and HD patients (FIGURE 4A, B, C and 4D). Only for ATP5I and UQCRH mRNA levels there was a statistically significant difference between CKD II–III and healthy subjects (FIGURE 4A and 4D, respectively). These results were in line with those obtained by the microarray analysis and indicated a possible similarity in oxidative phosphorylation system activity between CKD IV–V and HD patients.

### COXI and COXIV protein expression

**Figure 5:**
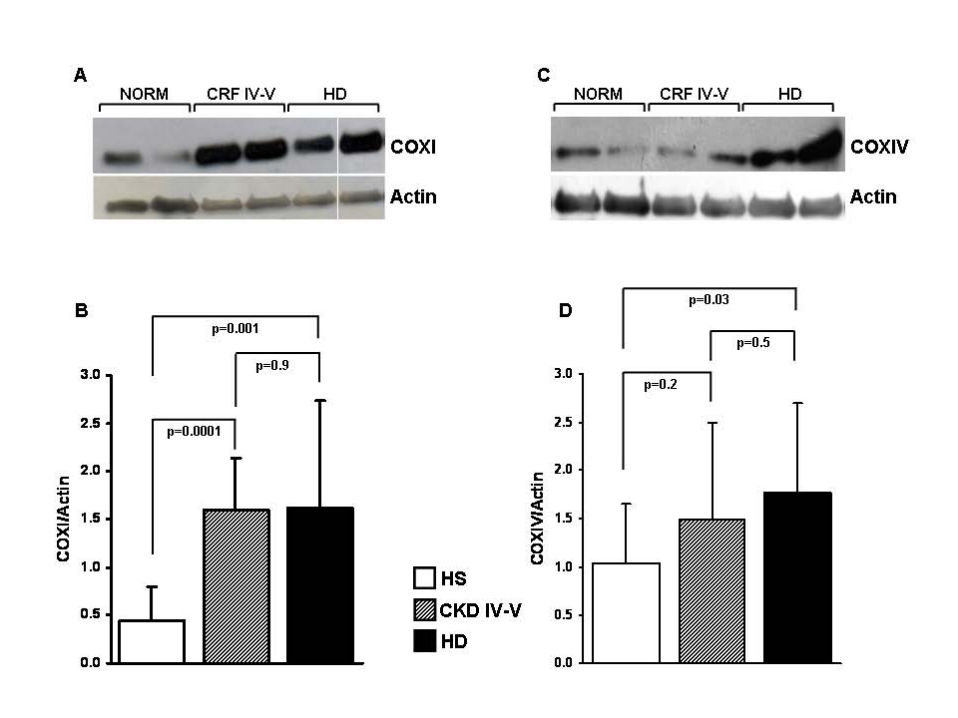
**COXI (A and B) and COXIV (C and D) protein expression in peripheral blood mononuclear cells (PBMC) from healthy subjects (HS), chronic kidney disease (CKD IV–V) and hemodialysis (HD) patients**. Panel A and C show a representative western blotting experiment respectively for COXI and COXIV. The histograms represent the mean ± SD of COXI (panel B) and COXIV (panel D) protein levels in total cell lysate of 12 HS, 10 CKD IV–V and 14 HD patients assessed by Western blotting. COXI and COXIV protein level was significantly higher in HD and CKD compared to HS.

Based on the results obtained in first part of the study, suggesting a significant involvement of the mitochondrial respiratory system in patients with a high degree of renal failure (CKD IV–V) and HD, we measured the protein level of the mitochondrial-encoded subunit I (COXI) and the nuclear-encoded subunit IV (COXIV) of complex IV in the testing-group. As shown in figure 5, COXI and COXIV levels were higher in CKD IV–V and HD compared to the control group. However, only for COXI, the comparison between CKD IV–V and healthy subjects reached the statistical significance. In addition, for both proteins, there were no significant differences in expression levels between CKD IV–V and HD patients.

### Complex IV (COX) activity

**Figure 6:**
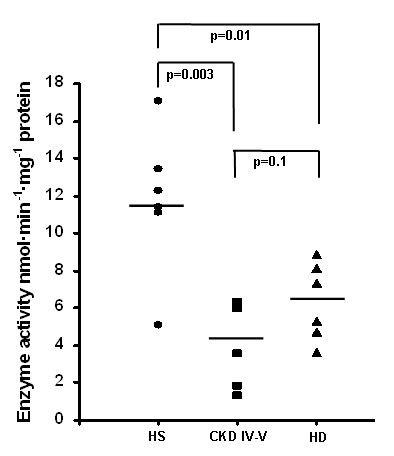
**Cytochrome c oxidase (COX) activity in peripheral blood mononuclear cells (PBMC) from healthy subjects (HS), chronic kidney disease (CKD IV–V) and hemodialysis (HD) patients**. Dot-plot represents the mean ± SD of COX enzymatic activity measured by following the oxidation of ferrocytochrome c at 550-540 nm in total cell lysate of 6 HS, 6 CKD and 6 HD. Activity was significantly lower in CKD IV–V and HD compared to HS. The line represents the mean value.

To obtain additional knowledge about the mitochondrial respiratory system function in patients with CKD, we measured the Complex IV activity in 6 HD, 6 CKD patients and 6 healthy subjects. Mean enzymatic activity was reduced by 65% in CKD IV–V and by 46% in HD patients compared to the control group, while no statistical difference was found in the comparison between CKD IV–V and HD patients (FIGURE 6).

### Intracellular Reactive Oxygen Species (ROS) levels

**Figure 7:**
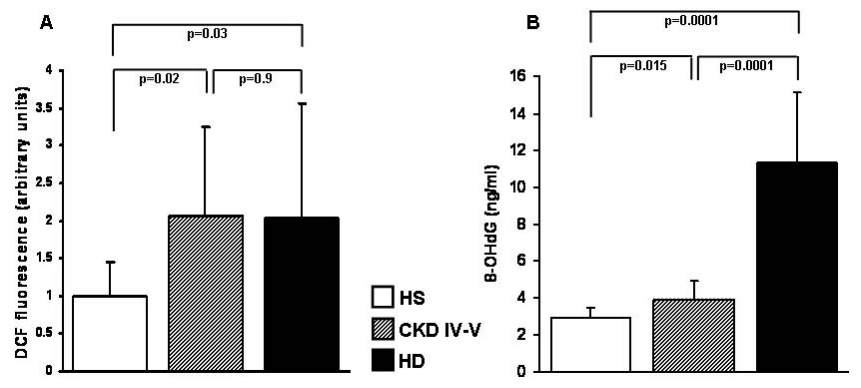
**Intracellular reactive oxygen species (ROS) and serum 8-hydroxydeoxyguanosine (8-OHdG) levels in healthy subjects (HS), chronic kidney disease (CKD IV–V) and hemodialysis (HD) patients**. The histograms represent the mean ± SD of 2',7'-Dichlorodihydrofluorescein (DCF) fluorescence (A) and serum 8-OHdG levels (B) in 12 HS, 10 CKD and 14 HD patients. DCF fluorescence in PBMC, reflecting the relative ROS levels, and the serum 8-OHdG levels were higher in HD and CKD compared to HS. In addition HD patients had higher 8-OHdG levels compared to CKD.

Since a significant part of ROS generation is dependent on the mitochondrial respiratory chain activity, we measured their levels in PBMC of all the testing-group population. Both, CKD IV–V and HD patients have significantly higher ROS levels compared to healthy subjects. No differences were observed between CKD IV–V and HD (FIGURE 7A).

### DNA oxidative damage

We measured serum 8-hydroxydeoxyguanosine (8-OHdG) levels in all testing-group population as an oxidative stress biomarker. Both, CKD IV–V and HD patients had significantly higher 8-OHdG levels compared to the control group. In addition HD patients showed higher 8-OHdG levels compared to CKD IV–V (FIGURE 7B).

## Discussion

In the last twenty years, several reports have focused on the molecular changes occurring during the onset and progression of CKD, but this rich literature appears fragmented and not exhaustive [10,12,21]. Analyzing these reports the attitude to focus on small number of biological elements and the lack of a comprehensive strategy to study the biochemical network associated with CKD is evident. In addition, despite the efforts of researchers and clinicians, CKD and renal replacement therapy are still associated to important clinical complications. In particular, during HD, the interaction of PBMC with dialytic membranes causes their activation with a consequent increased synthesis and release of pro-inflammatory cytokines [9-11] and imbalance between pro- and anti-oxidant activities, resulting in high oxidative stress with elevated synthesis of ROS [13,14].

In the present study, we used a whole genome analysis by microarray technology, combined with classical biomolecular approaches, to detect unrecognized biological elements deregulated in subjects with CKD and to identify new potential targets for pharmacological interventions. Microarray analysis revealed a specific genomic fingerprint able to identify HD and CKD patients from healthy subjects. Functional analysis by IPA and KEGG demonstrated that 25% of the selected genes encodes for protein involved in mitochondrial oxidative phosphorylation system.

Mitochondria are essential eukaryotic cells organelles involved in several metabolic pathways, calcium and iron homeostasis, ROS production and programmed cell death [28]. They present an outer and inner membrane, the latter of which would be impermeable to all molecules in the absence of specific carriers and contains the enzymatic oxidative phosphorylation complex. The respiratory flux is due to the donation of electrons from NAD- or FAD-dependent substrates, via respiratory chain, to molecular oxygen which is finally reduced to water. Simultaneously, the energy conserving complexes I, III and IV build up a trans-membrane electrochemical gradient by coupling the electron transfer activity to proton translocation from the matrix to the outer side of the inner mitochondrial membrane. Complex V utilizes backward the electrochemical gradient for ATP synthesis.

A significant number of the genes discriminating CKD and HD patients from healthy subjects were involved in the synthesis of important nuclear-encoded structural subunits of the oxidative phosphorylation complexes. In particular, *NDUFA6*, *NDUFS5*, *NDUFA1 *and *NDUF*B1 encode for subunits of the Complex I (NADH dehydrogenase) that is involved in the transfer of electrons from NADH to ubiquinone [29]. Interestingly, another gene encoding for a subunit of this complex (*NDUFA2*) was identified by microarray analysis being up-regulated in skeletal muscle biopsy specimens of HD patients compared to control subjects without renal failure [27]. *COX6C*, *COX7C *encode for two subunits of the cytochrome c oxidase (COX or Complex IV), the terminal enzyme of the mitochondrial respiratory chain catalyzing the electron transfer from reduced cytochrome c to oxygen [30]. *ATP5O*, *ATP5I *and *ATP5J *encode for components of the ATP synthase (complex V).

To investigate whether the increased gene expression observed in our genomic study was, indeed, associated with an increased oxidative phosphorylation system activity, we analyzed the protein expression of mitochondrial-coding COXI (catalytic subunit) and nuclear-coding COXIV subunits of Complex IV. Although these two essential subunits of Complex IV have not been identified by our genomic approach, we decided to measure their levels based on the evidence that COX exerts a tight control on the respiration of a variety of human cells including myeloma [31] and Jurkat blood cells [32]. The observation that the expression of both proteins was higher in HD and CKD patients compared to healthy subjects further confirmed the data obtained by the microarray analysis.

Additionally, when we measured the activity of complex IV, despite the expected high interindividual variability [33], we observed a dramatic reduction in both CKD and HD patients compared to healthy subjects. This is in line with experimental evidences suggesting that chronic oxidative stress and oxidant injury may enhance the expression of several nuclear mitochondrial biogenesis genes [34,35]. Indeed, our results highlight the role of oxidative stress in this process since both CKD and HD patients were characterized by an elevated intracellular ROS production and a high level of 8-OHdG, a marker of oxidative stress to DNA [36]. ROS may deeply influence a variety of key cell functions damaging proteins, lipids and nucleic acids [37-39] and inhibiting directly the enzymatic activities of several elements of the cellular respiratory chains [40-42]. Thus, our hypothesis is that an increased production of ROS due to the effect of pro-inflammatory mediators may cause a profound inhibition of the oxidative phosphorylation system leading to a compensatory "hypertrophy" of its components. In addition, a hypertrophic and impaired oxidative phosphorylation system may prime a vicious circle, causing a continuous release of ROS.

An interesting point in our study is that CKD and HD patients are virtually undistinguishable when it comes to the expression of oxidative phosphorylation system components. On this basis, we may consider this finding as a genomic hallmark of CKD itself that is not modulated by HD treatment. However, it should be taken into consideration that our HD patient's population has been treated with highly biocompatible synthetic membranes, previously shown to cause a very limited lymphomononuclear cell activation [43-45], although, Raj DS et al. have reported that mitochondrial dysfunction is induced even with the use of biocompatible membrane [46].

## Conclusion

In conclusion, our results suggest, for the first time, a clear deregulation of the mitochondrial respiratory machinery in the CKD patients closely associated with an enhanced oxidative stress. This may explain previous fragmented reports indicating a subnormal energy metabolism in this complex population. Finally, our research strategy may delineate a new methodological approach for biologists and clinicians who may collaborate and achieve what has been recently termed as "translational medicine".

## Methods

### Patients

A total of 80 subjects, after signing informed consent according to declaration of Helsinki, were included in the study and divided in a training-group (n = 44) and a testing-group (n = 36).

#### A) Training-group

This population was used for the microarray analysis and to generate the initial genomic model. It included 8 healthy subjects, 9 CKD patients on stage II–III (mean ± SD of estimated GFR by MDRD formula: 41.4 ± 4.3 ml/min) and 17 HD patients. To define the influence of the degree of renal failure on the transcriptomic profile and to better select the population for the second part of the study, we added 10 CKD patients on stage IV–V (mean ± SD of estimated GFR by MDRD formula: 19.8 ± 3.6 ml/min) in the RT-PCR experiments.

#### B) Testing-group

This population was used to confirm the hypothesis generated by the training-group and it included 12 healthy subjects, 10 CKD IV–V (mean ± SD of estimated GFR by MDRD formula: 20.2 ± 3.7 ml/min) and 14 HD patients. In this part of the study, we did not include CKD patients on stage II–III based on the results obtained in the training-group showing only a slight difference in gene expression between this group and the control group.

All HD patients were stably treated, for at least 1 year, three times a week (4–5 hours per session), using synthetic membrane dialyzers. During the study period, no CKD patients received dialysis treatment. In addition, all patients suffering from infectious diseases, diabetes, chronic lung diseases, neoplasm, or inflammatory diseases and patients receiving antibiotics, corticosteroids, or non-steroidal anti-inflammatory agents were excluded. No patients had symptomatic coronary artery diseases or a family history of premature cardiovascular diseases. The main clinical and demographic characteristics of the subjects included in the training and testing group are summarized in TABLE 1 and 2, respectively.

**Table 1:** Patient demographics and clinical characteristics of the training-group

	**healthy subjects**	**CKD II–III**	**CKD IV–V**	**HD**	***P-value***
Number	8	9	10	17	/
Gender (M/F)	3/5	5/4	6/4	10/7	*n.s.*
Age (years)	49.37 ± 10.68	49.12 ± 9.82	51.11 ± 7.18	52.86 ± 8.63	*n.s.*
Cause of CKD: GN, ADPKD, renal vascular disease, unknown	/	3,2,1,3	4,2,2,2	6,4,2,5	*n.s.*
Time on dialysis (years)	/	/	/	3.98 ± 0.56	/
BMI (kg/m^2^)	22.9 ± 1.42	22.1 ± 0.92	21.3 ± 1.05	21.8 ± 1.01	*p = 0.02*
Systolic blood pressure (mmHg)	121 ± 3.51	135 ± 17.4	137 ± 13.22	136 ± 13.22	*p = 0.05*
Diastolyc blood pressure (mmHg)	72.12 ± 7.59	83.11 ± 6.23	82 ± 9.54	84 ± 10.01	*p = 0.02*
Total protein (g/dl)	7.02 ± 0.31	6.60 ± 0.42	5.82 ± 0.72	5.96 ± 0.36	*p < 0.01*
Albumin (g/dl)	4.4 ± 0.11	4.1 ± 0.24	3.93 ± 0.67	3.48 ± 0.51	*p < 0.01*
Ferritin (ng/ml)	87 ± 40.1	136 ± 56.1	376 ± 318.2	451 ± 283.1	*p < 0.01*
Hemoglobin (g/dl)	13.65 ± 1.17	12.07 ± 1.70	11.13 ± 1.01	11.48 ± 1.98	*p *< 0.01

GN: Glomerulonephritis; ADPKD: Autosomal dominant polycystic kidney disease. Values are expressed as mean ± SD. *P-values *calculated by ANOVA.

**Table 2:** Patient demographics and clinical characteristics of the testing-group

	**healthy subjects**	**CKD IV–V**	**HD**	***P-value***
Number	12	10	14	/
Gender (M/F)	7/5	6/4	8/6	*n.s.*
Age (years)	50.78 ± 10.45	51.64 ± 10.26	52.86 ± 8.63	*n.s.*
Cause of CKD: GN, ADPKD, renal vascular disease, unknown	/	4,2,2,2	5,3,1,5	*n.s.*
Time on dialysis (years)	/	/	3.98 ± 0.56	/
BMI (kg/m^2^)	23.1 ± 0.82	21.9 ± 0.74	22.1 ± 1.01	*p < 0.01*
Systolic blood pressure (mmHg)	120 ± 4.01	136 ± 9.71	138 ± 8.11	*p < 0.01*
Diastolyc blood pressure (mmHg)	74.36 ± 5.31	82.1 ± 10.03	88 ± 10.86	*p < 0.01*
Total protein (g/dl)	6.98 ± 0.41	5.98 ± 0.76	6.02 ± 0.31	*p < 0.01*
Albumin (g/dl)	4.3 ± 0.31	3.71 ± 0.42	3.48 ± 0.51	*p < 0.01*
Ferritin (ng/ml)	79 ± 62.3	210 ± 183.6	451 ± 283.1	*p < 0.001*
Hemoglobin (g/dl)	13.46 ± 1.62	11.88 ± 1.87	11.48 ± 1.98	*p < 0.01*

GN: Glomerulonephritis; ADPKD: Autosomal dominant polycystic kidney disease. Values are expressed as mean ± SD. *P-values *calculated by ANOVA.

### PBMC isolation

Twenty ml of whole blood were collected from all subjects included in both training- and testing-group. For HD patient the biological material was obtained at the beginning of the second HD session of the week. PBMC were isolated by density separation over a Ficoll-Paque™ (GE healthcare, Sweden) gradient (460 g for 30 min). PBMC were washed three times with PBS pH 7.4/1 mM EDTA (Sigma, Milan, Italy). Cells were then counted and their viability was assessed by trypan blue exclusion (>90% PBMC were viable).

### RNA extraction and gene expression profiling

For all subjects included in the training-group, total RNA was isolated by RNeasy mini kit Qiagen (QIAGEN AG, Basel, Switzerland) from a minimum of 5 × 10^6 ^cryopreserved PBMC. RNA was, then, processed and hybridized to the GeneChip Human Genome U133 oligonucleotide microarray (n = 5 to the HG-U133-Plus and n = 29 to the HG-U133A array). For our analysis, we used a dataset including 22,283 gene probe sets, representing 12,357 human genes and 3,800 ESTs (Affymetrix; see the manufacturer's manual for detailed protocol). We used the default settings of Affymetrix Microarray Suite software version 5 to calculate scaled gene expression values. Results of the microarray experiments are available in Gene Expression Omnibus (Accession number GSE15072).

### Reverse transcription-polymerase chain reaction (RT-PCR)

Reverse transcription of RNA was performed using the High Capacity cDNA Reverse Transcription Kit (Applied Biosystems), following the manufacture's instructions. One μg of RNA was reverse transcribed using random primer and MultiScribe Reverse Transcriptase. All amplification reactions were performed using primer designed by the aid of the Primer3 software (). For COX6C mRNA expression, the primer sequences were: forward 5'-ctttgtataagtttcgtgtgg-3' and reverse 5'-attcatgtgtcatagttcagg-3'. The conditions of amplification were: 94°C for 20 sec, 58°C for 20 sec, 70°C for 20 sec for a total of 35 cycles of amplification. The primers used for COX7C were forward 5'-ccctgggaagaatttgcca-3' and reverse 5'-ggaactgaaacatccttatg-3'. The conditions of amplification were: 94°C for 20 sec, 56°C for 20 sec, 70°C for 20 sec for a total of 30 cycles of amplification. The primers used for ATP5I were forward 5'-cgctacaattacctaaaacctc-3' and reverse 5'-ctttattcatccgctgctggt-3'. The conditions of amplification were: 94°C for 20 sec, 60°C for 20 sec, 70°C for 20 sec for a total of 30 cycles of amplification. The primers used for UQCRH were forward 5'-agggaccattgcgtggcc-3' and reverse 5'-agctaccagcctaagccaaa-3'. The conditions of amplification were: 94°C for 20 sec, 58°C for 20 sec, 70°C for 20 sec for a total of 30 cycles of amplification. *β*-actin PCR products were used as control gene. *β*-actin primer sequences were forward 5'-ggcatcgtgatggactccg-3' and reverse 5'-gctggaaggtggacagcga-3'. The conditions of amplification were: 94°C for 20 sec, 65°C for 20 sec, 70°C for 20 sec for a total of 30 cycles of amplification. PCR products were electrophoretically separated on agarose gels and stained with ethidium bromide. The density of each band, corresponding to a specific PCR product, was densitometrically quantified by pixel density using NIH Image J image software . The ratio between COX6C, COX7C, ATP5I, UQCRH and β-actin PCR products were used as indexes of COX6C, COX7C, ATP5I, UQCRH gene expression.

### Western blot analysis

For western blots, equal amounts of total cellular proteins (10–20 μg) extracted by PBMC isolated from all patients included in the testing-group, were separated on a 13% SDS-polyacrylamide gel and transferred onto nitrocellulose membrane. Membranes were then blocked with 5% non-fat milk in 0.1% TBS-Tween-20 and probed with specific antibodies against the mitochondrial-encoded subunit I (COXI gene, Molecular Probes, Eugene, OR) and the nuclear-encoded subunit IV (COXIV, Molecular Probes) of respiratory complex IV. Membranes were also probed with specific antibody against actin (from Sigma) utilized as loading control. Membranes were finally incubated with HRP-conjugated secondary antibodies and developed with Immune-Star HRP chemiluminescent kit (Bio-Rad). The specific COXI and COX IV bands were quantified by pixel density using NIH ImageJ image software and normalized to the actin band.

### Assessment of COX IV activity

PBMC, isolated by 6 HD, 6 CKD IV–V and 6 healthy subjects, were resuspended in hypotonic medium (25 mM potassium phosphate pH 7.4, 5 mM MgCl_2_) and supplemented with a protease inhibitors cocktail (Roche Diagnostics, Mannheim, Germany). In order to allow complete accessibility of substrates to the inner mitochondrial membrane enzymes, samples were freeze-thawed three times. Protein concentration was determined according to Bradford method, using BSA as standard. The enzyme activity was measured at 30°C using 0.03–0.1 mg/ml of total cell proteins using a Beckman DU7400 spectrophotometer equipped with a rapid-mixing apparatus, essentially as previously described [47].

The KCN-sensitive cytochrome c oxidase (complex IV) activity was measured by following the initial rate of oxidation of ferrocytochrome c (20 μM) at 550-540 nm (Δε = 19.1 mM^-1^cm^-1^).

### Measurement of intracellular ROS production

For all patients included in the testing-group, peroxide-sensitive fluorescent probe 2',7'-dichlorodihydrofluorescin diacetate (Molecular Probes) was used to assess the generation of intracellular peroxide (hydrogen peroxide and peroxynitrite) as previously described [48]. This compound is converted by intracellular esterases to 2',7'-dichlorodihydrofluorescein, which is then oxidized by hydrogen peroxide to the highly fluorescent 2',7'-dichlorodihydrofluorescein (DCF). PBMC were maintained in culture medium (RPMI-1640, Sigma, Milan, Italy) supplemented with 10% fetal bovine serum (FBS), 1% penicillin-streptomycin, 2 mM L-glutamine for 24 hours. The cells were, then, washed with Hank's balanced salt solution (HBSS) without phenol red and incubated with 5 μM 2',7'-dichlorodihydrofluorescin diacetate in the dark for 20 min at 37°C. Subsequently, DCF fluorescence was detected at excitation and emission wavelengths of 488 nm and 520 nm, respectively, and measured with a fluorescence reader (JASCO-FP-6200).

### 8-OHdG assay

Serum 8-OHdG levels were measured with a competitive enzyme-linked immunosorbent assay kit (high sensitivity 8-OhdG Check; Japan Institute for the Control of Aging, Shizuoka, Japan) in all subjects included in the testing-group. Briefly, 50 μl of sample and 8-OHdG monoclonal antibody were added to each well and incubated at 4°C overnight. After washing, horseradish peroxidase-conjugated antibody was added to the plate and incubated for 60 min. The plate was washed again and 100 μl of enzyme substrate was added to each well and incubate at room temperature for 15 minutes. After terminating the reaction the absorbance was read at 450 nm.

### Statistical analysis

Results were expressed as mean ± SD. *t*-test, ANOVA and Fisher's exact test were used to assess differences in clinical and demographic features. A value of *P *< 0.05 was considered to be statistically significant.

For microarray analysis, gene expression values for the 22,283 gene probe sets, scaled to the target intensity of 2,500, were log transformed. ANOVA and permutation analysis by Storey's q-value [49] were used to identify gene probe sets discriminating CKD and HD patients from healthy subjects. However, to better select genes deregulated in patients with advanced renal failure and to reduce the number of variables to include in the second part of the study, we decided to focus our attention on the top up-regulated gene probe sets in HD patients. R 2.0.1 statistical software was used to perform the above analyses. Hierarchical clustering was build using Spotfire DecisionSite 9.0 .

To assess the biological relationships among genes, we used the Ingenuity Pathway Analysis software (IPA, Ingenuity System, Redwood City, CA; ) and the Kyoto Encyclopedia of Genes and Genomes (KEGG, ). IPA is a knowledge database generated from the peer-reviewed scientific publications that enables discovery, visualization and exploration of functional biological networks in gene expression data and delineates the functions most significant to those networks. A score is assigned to each generated network according to the number of differentially regulated focus genes in our dataset. These scores are derived from negative logarithm of the *P *indicative of the likelihood that focus genes found together in a network due to random chance. Scores of 4 or higher have 99.9% confidence level of significance. KEGG is a collection of online databases dealing with genome, enzymatic pathways and biological chemicals. KEGG connects known information on molecular interaction networks, such as pathways and complexes, about genes and proteins generated by genome projects and biochemical compounds and reactions.

## Authors' contributions

SG and SS carried out the biomolecular experiments. GZ, PP and MC performed microarray experiment and data analysis. GV designed the experimental part to study the mitochondrial activity. DL carried out complex IV activity assay. GG and GP helped in the manuscript writing and data analysis. FPS supervised microarray part of the study and helped to write the paper. All authors read and approved the final manuscript.
